# Infectious Diseases: A Geographic Guide, Second Edition

**DOI:** 10.3201/eid2503.181500

**Published:** 2019-03

**Authors:** Julian W. Tang

**Affiliations:** University Hospitals of Leicester National Health Service Trust, Leicester, UK

**Keywords:** infectious diseases, geographic guide, travel medicine, book review

In these modern times of frequent overseas travel, a succinct reference describing infectious hazards for each potential destination can prompt travelers to seek preventive medical advice before they travel, rather than treating infections after they return. This second edition of Infectious Diseases: A Geographic Guide (Figure) makes a useful attempt at providing a resource for travelers and clinicians alike.

**Figure:**
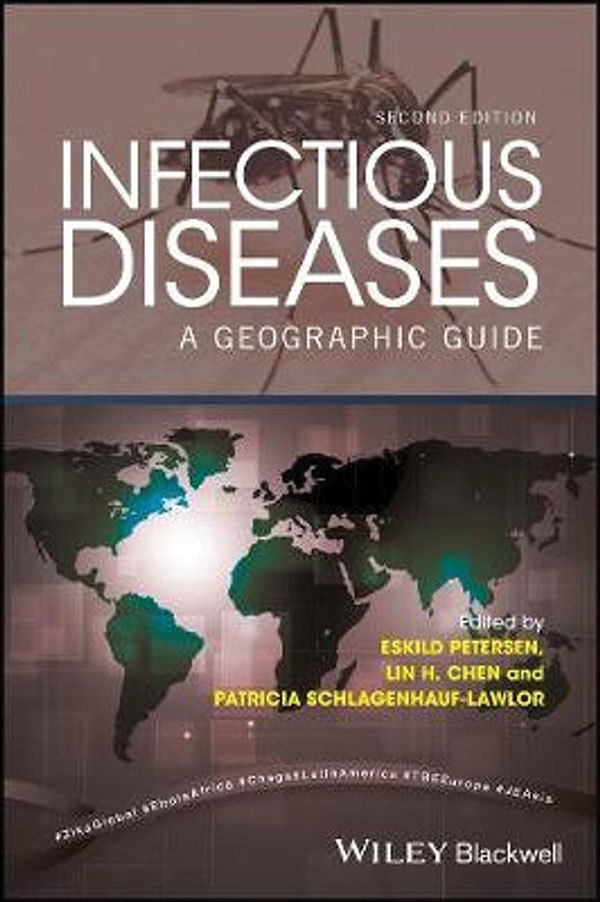
Infectious Diseases: A Geographic Guide, Second Edition

The volume is divided into 3 sections. The first introduces concepts related to travel-associated infections. The second, main section includes geographic regions and descriptions of infectious agents endemic to each. The closing chapters provide additional information, such as the impact of migration and climate change on the distribution of infectious agents.

The introductory chapters vary in style, depth, and length; cover some of the history and politics of infectious diseases; and offer insight into the resources and technology required to detect and combat pathogens. Chapter 6, in particular, gives a fairly detailed view of diagnostic tests and lays out the anatomic approach the authors use to describe disease symptoms in the later geographic chapters. Written by the lead editor, Eskild Petersen, this chapter not only explains and compares the types and limitations (e.g., sensitivity and specificity) of diagnostic tests required for detecting the various pathogens but also the accompanying characteristics of other tests (e.g., biochemistry, cytology, radiological imaging) routinely requested when investigating these infections.

The chapters in the geographic section are the focus of the text. Each follows a formula that starts with a boxed summary, then progresses through a head-to-toe anatomic listing of pathogens and associated symptoms in each organ, mostly in the form of tables indicating frequency of occurrence. The authors added a nice touch by further separating some tables into infections with symptoms lasting >4 weeks or <4 weeks, which can help clinicians narrow the differential diagnosis. Each chapter also includes sections describing antibiotic-resistant and vaccine-preventable infections.

Chapters in the geographic section necessarily vary in length and depth depending on the amount of data available, but there are a few unexpected omissions. For instance, plague (*Yersinia pestis*) is absent from the chapters on East Africa and North America, despite being endemic in Madagascar and the United States for more than a century. In addition, severe fever with thrombocytopenia syndrome, an emerging infectious disease identified in China, is absent from the chapter on East Asia. Surprisingly, the index is relatively inconsistent, making it important to read both the accompanying text and the tables. For instance, the East Asia chapter only includes avian influenza H5N1 in the index, not avian influenza H7N9, but the authors discuss only the latter briefly in the text. One table in the North America chapter lists influenza as a rare microorganism under cardiopulmonary infections, which is clearly inaccurate. An additional column devoted to seasonal pathogens might remedy similar inaccuracies in some tables.

This text mostly achieves what the authors propose, although the content of some chapters needs updating. In addition, a more detailed and comprehensive index, ideally separate for the tables and main text, would make it even more helpful for busy clinicians. Overall, this is a useful companion for both the physician and traveler alike.

